# Supplementary material for: Soil moisture dominates dryness stress on ecosystem production globally
Source: Nat Commun. 2020 Sep 29;11:4892. doi: 10.1038/s41467-020-18631-1 (PMC7524720; doi:10.1038/s41467-020-18631-1)
Supplement: Supplementary file 1 — supplementary information [file 41467_2020_18631_MOESM1_ESM.pdf]

Supplementary Information for "Soil moisture dominates dryness stress on ecosystem production globally" by Liu et al.

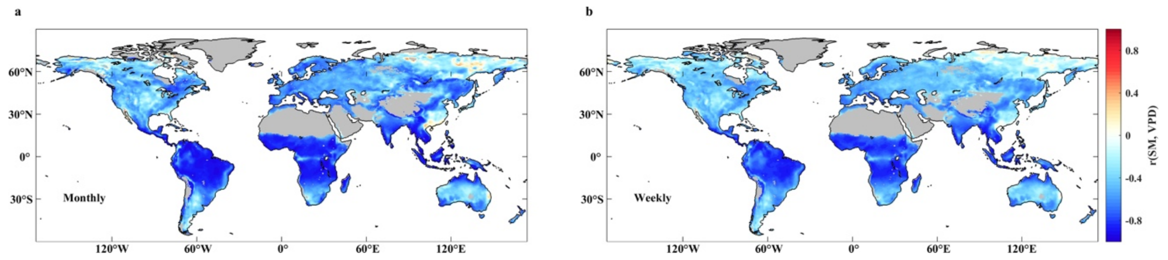

Supplementary Figure 1. Spatial distribution of Pearson's correlation coefficient between SM and VPD at (**a**) monthly and (**b**) weekly scale. Regions with sparse vegetation and regions without valid data are masked in gray.

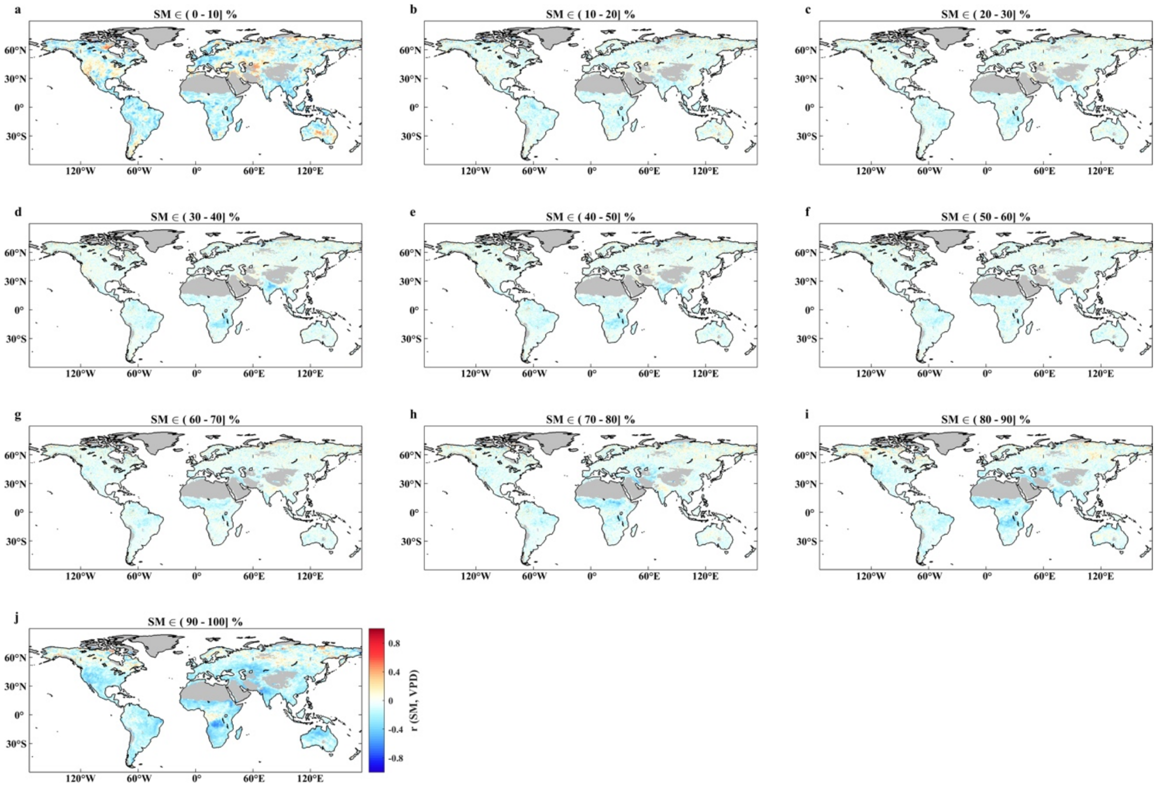

Supplementary Figure 2. Spatial distribution of Pearson's correlation coefficient between SM and VPD (i.e., r(SM, VPD)) in (**a-j**) 0th-10th, 10th-20th, …, 80th-90th, and 90th-100th percentiles of SM. Regions with sparse vegetation and regions without valid data are masked in gray.

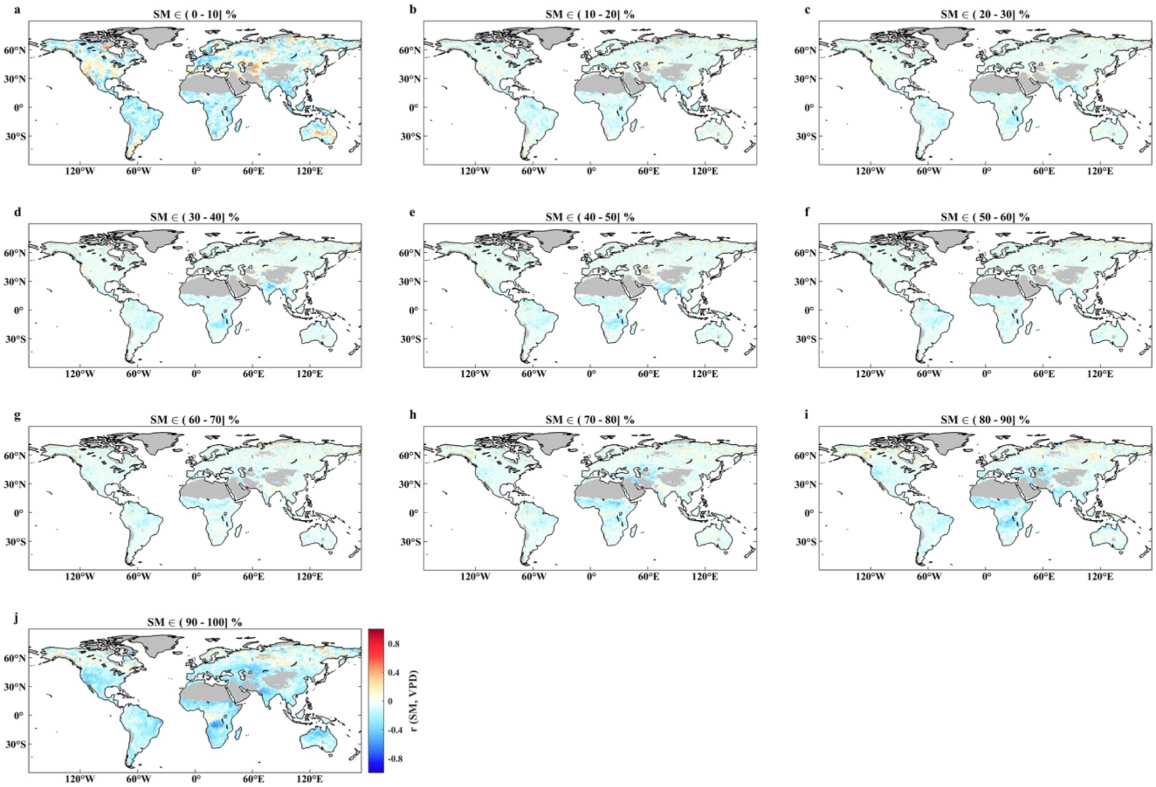

Supplementary Figure 3. Spatial distribution of Pearson's correlation coefficient between SM and VPD (i.e., r(SM, VPD)) in (**a-j**) 0th-10th, 10th-20th, …, 80th-90th, and 90th-100th percentiles of VPD. Regions with sparse vegetation and regions without valid data are masked in gray.

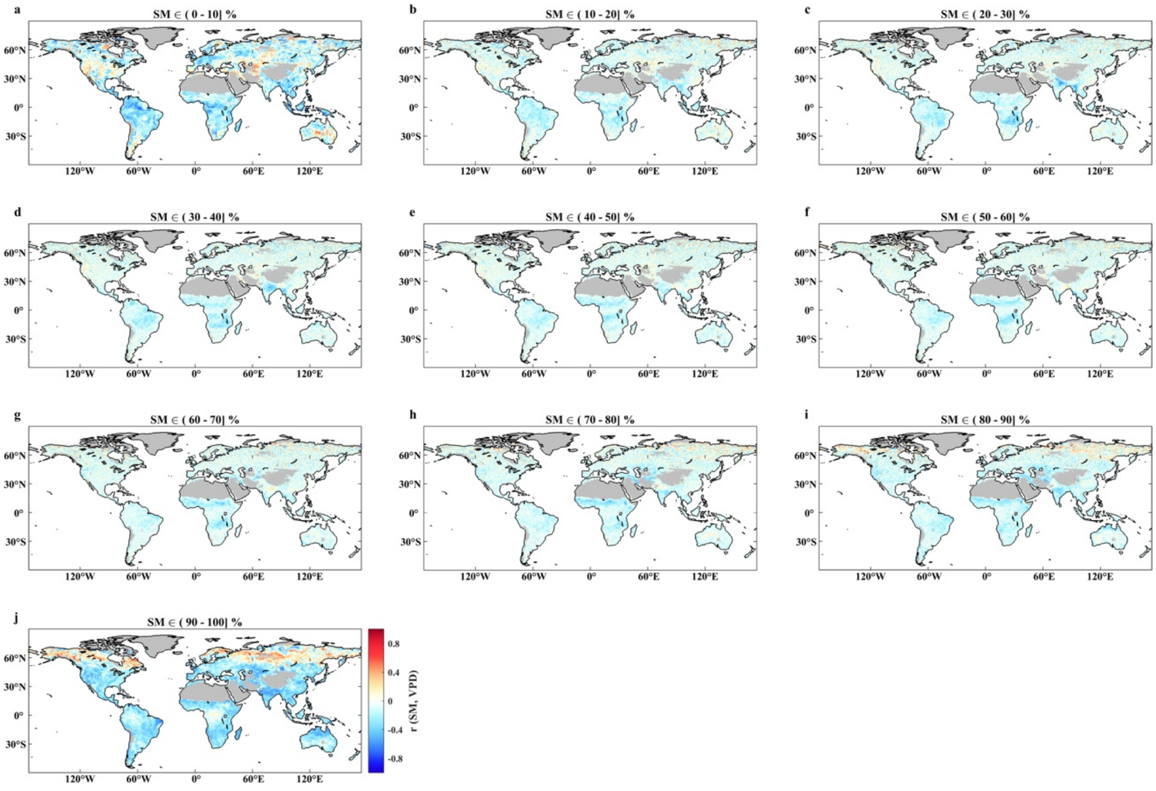

Supplementary Figure 4. Spatial distribution of Pearson's correlation coefficient between SM and VPD (i.e., r(SM, VPD)) in (**a-j**) 0th-10th, 10th-20th, …, 80th-90th, and 90th-100th percentiles of SM in 4-day bins. Regions with sparse vegetation and regions without valid data are masked in gray.

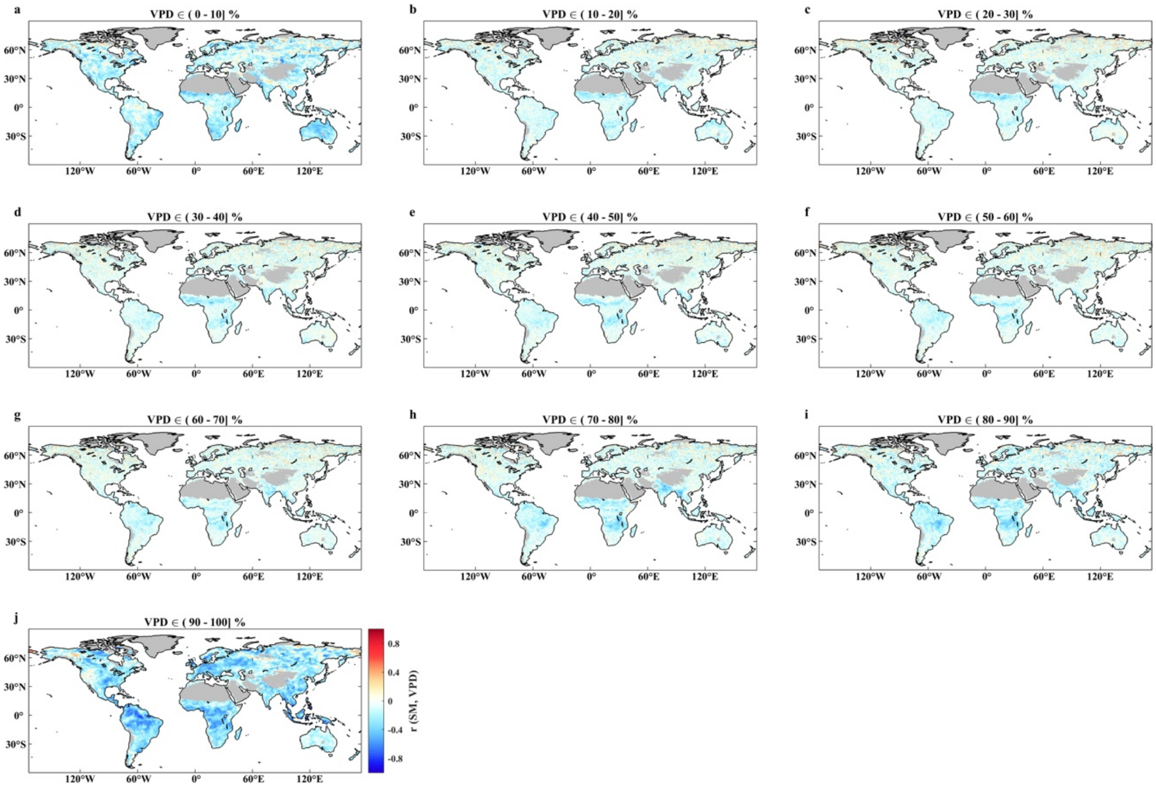

Supplementary Figure 5. Spatial distribution of Pearson's correlation coefficient between SM and VPD (i.e., r(SM, VPD)) in (**a-j**) 0th-10th, 10th-20th, …, 80th-90th, and 90th-100th percentiles of VPD in 4-day bins. Regions with sparse vegetation and regions without valid data are masked in gray.

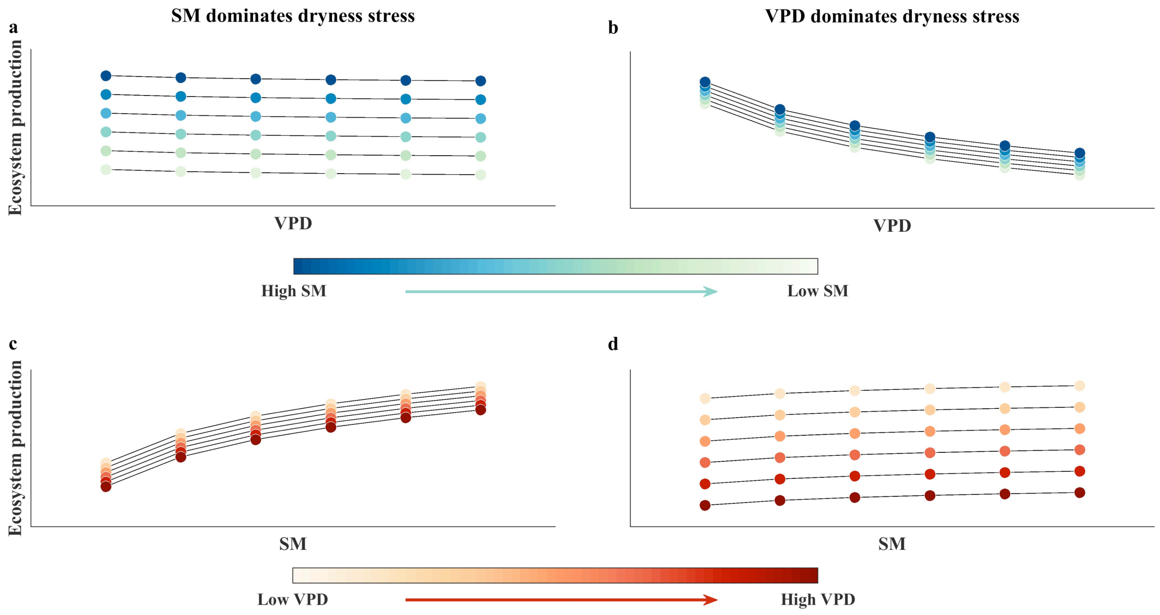

Supplementary Figure 6. Conceptual illustration of ecosystem productivity response to dryness under the condition of different dominating drivers. **a, c**, If SM dominates the dryness stress, higher VPD would not lead large decrease in ecosystem production, while lower SM can depress ecosystem production regardless of VPD variations. **b, d**, If VPD dominates the dryness stress, lower SM would not lead large decrease in ecosystem production, while higher VPD can depress ecosystem production regardless of SM variations.

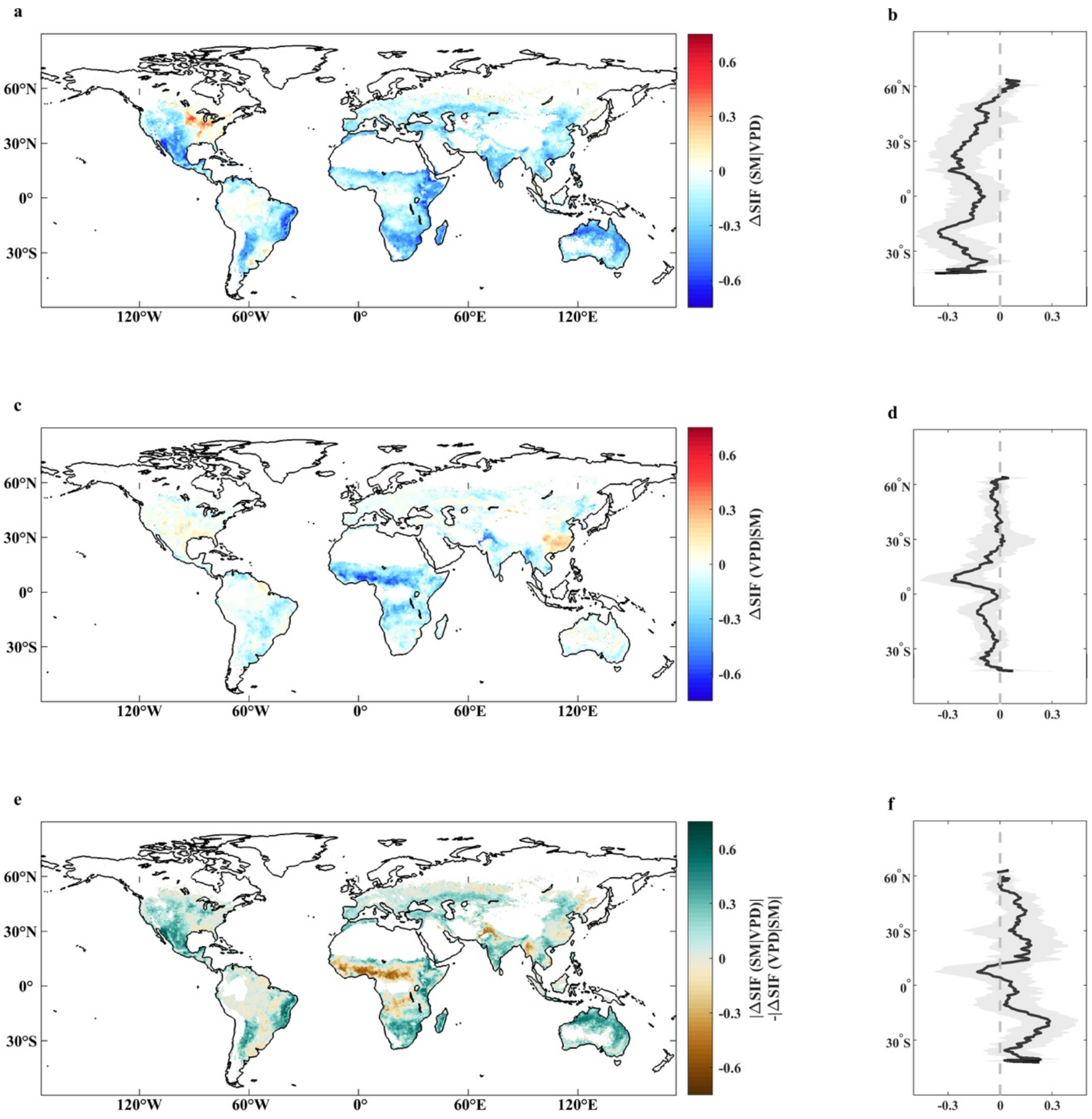

Supplementary Figure 7. Same as Fig. 4, but using MERRA-2 soil moisture and MERRA-2 VPD. Black lines indicate the mean values, and gray shaded bands show the standard deviation.

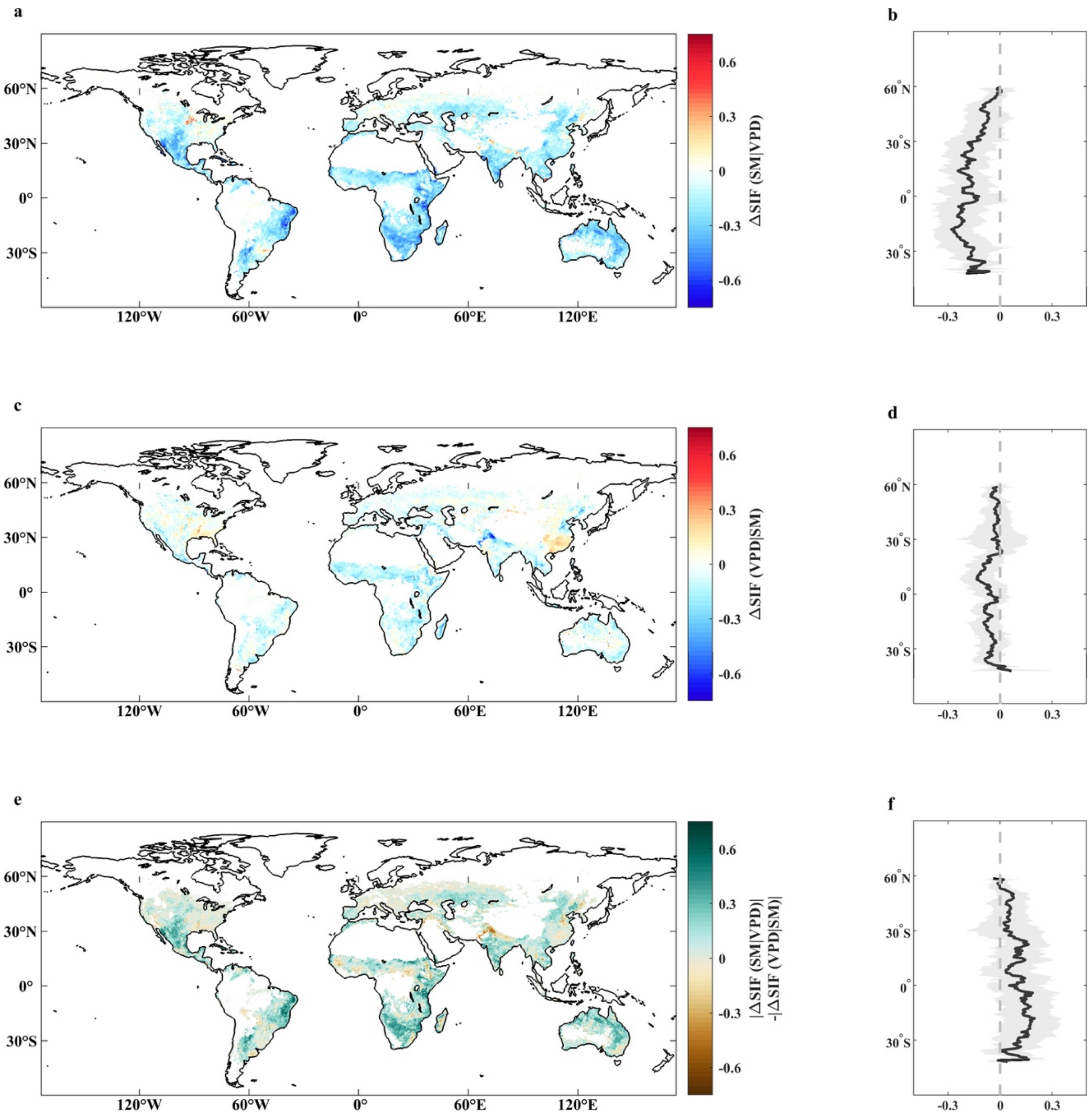

Supplementary Figure 8. Same as Fig. 4, but using ESA CCI soil moisture and ERA-Interim VPD. Black lines indicate the mean values, and gray shaded bands show the standard deviation.

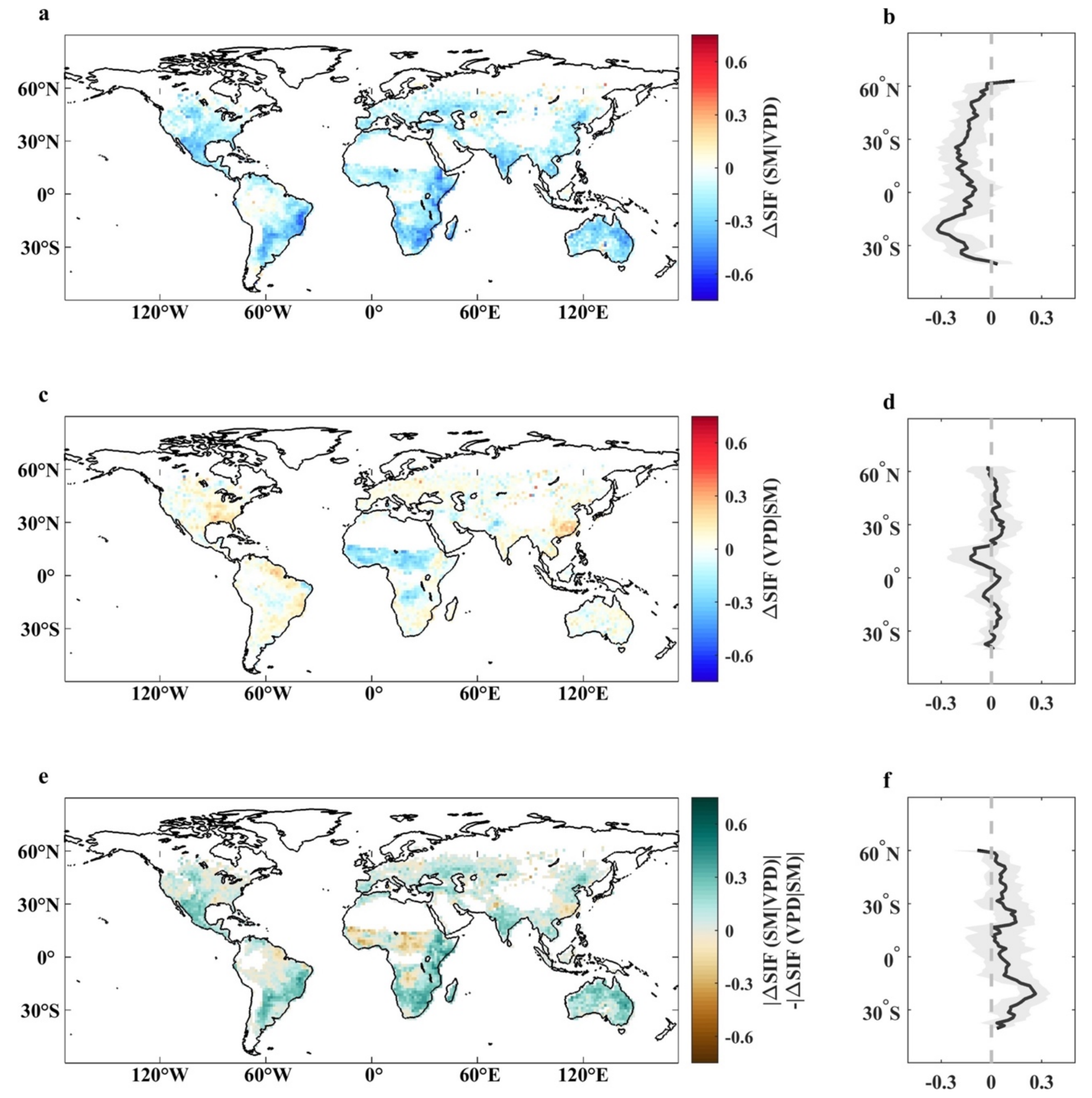

Supplementary Figure 9. Same as Fig. 4, but using the daily mean SCIAMACHY SIF and ERA-Interim soil moisture and VPD. Black lines indicate the mean values, and gray shaded bands show the standard deviation.

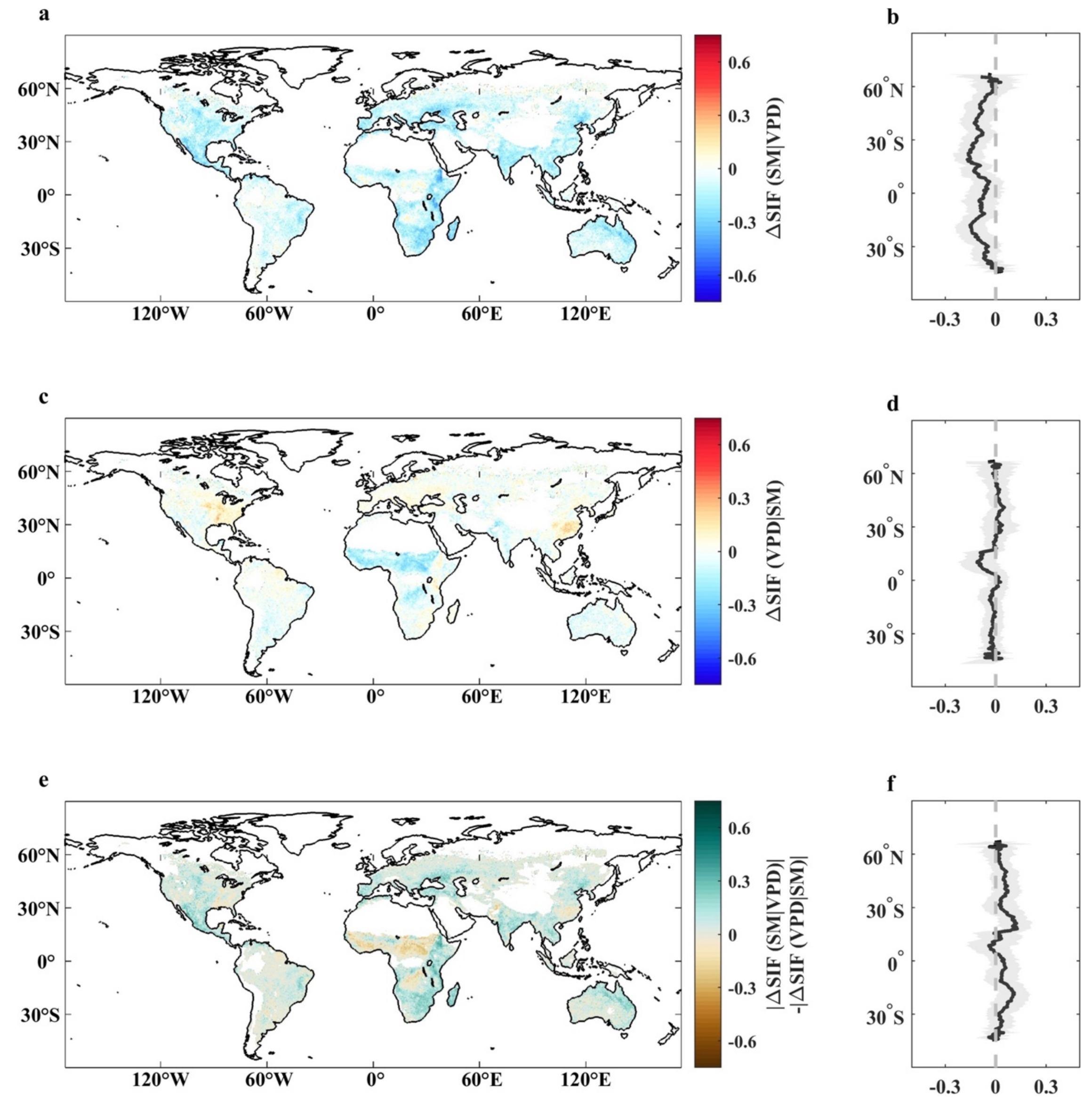

Supplementary Figure 10. Same as Fig. 4, but using the daily mean GOME-2 N28 SIF and ERA-Interim soil moisture and VPD. Black lines indicate the mean values, and gray shaded bands show the standard deviation.

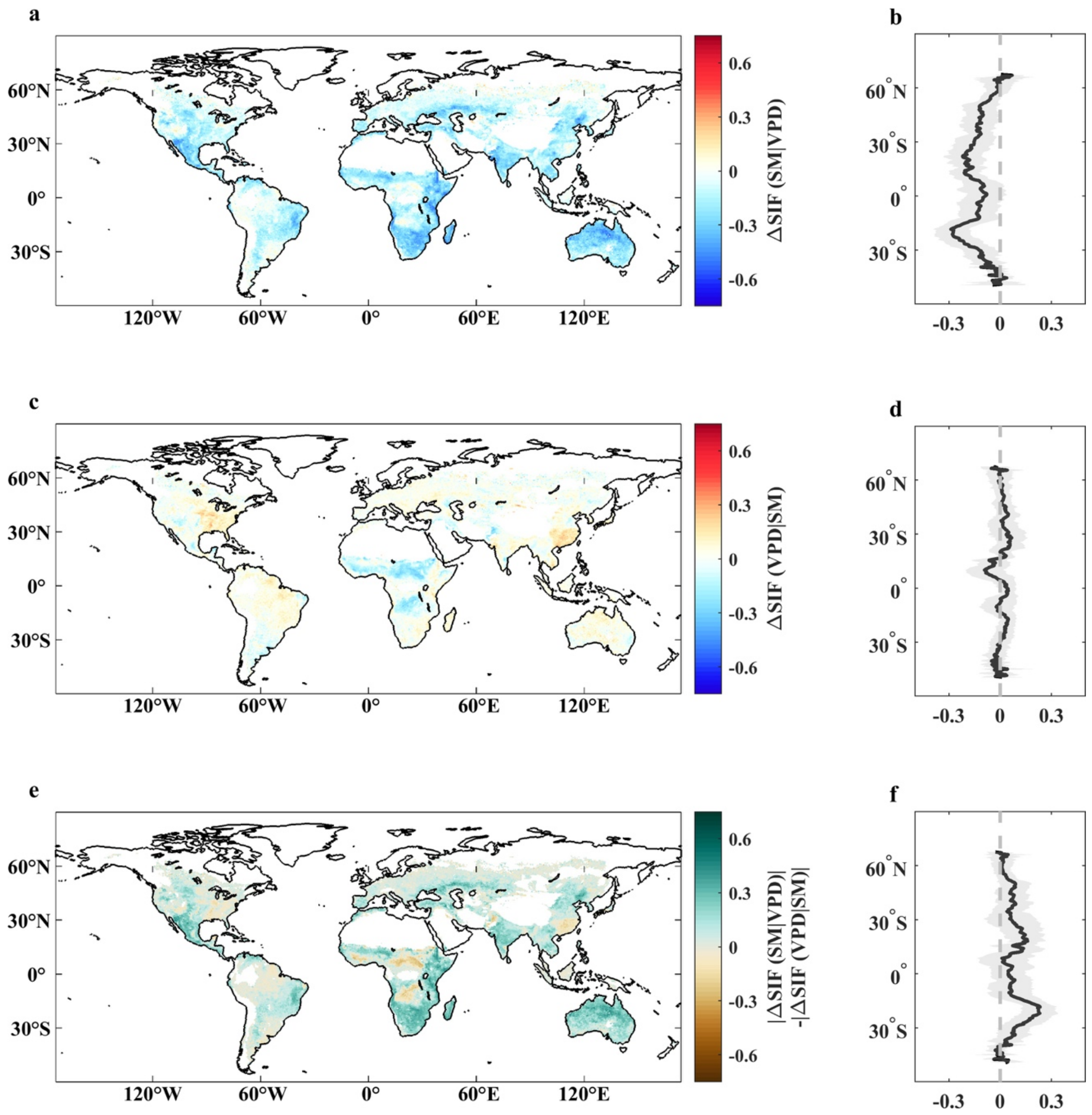

Supplementary Figure 11. Same as Fig. 4, but using the daily mean GOME-2 GFZ SIF and ERA-Interim soil moisture and VPD. Black lines indicate the mean values, and gray shaded bands show the standard deviation.

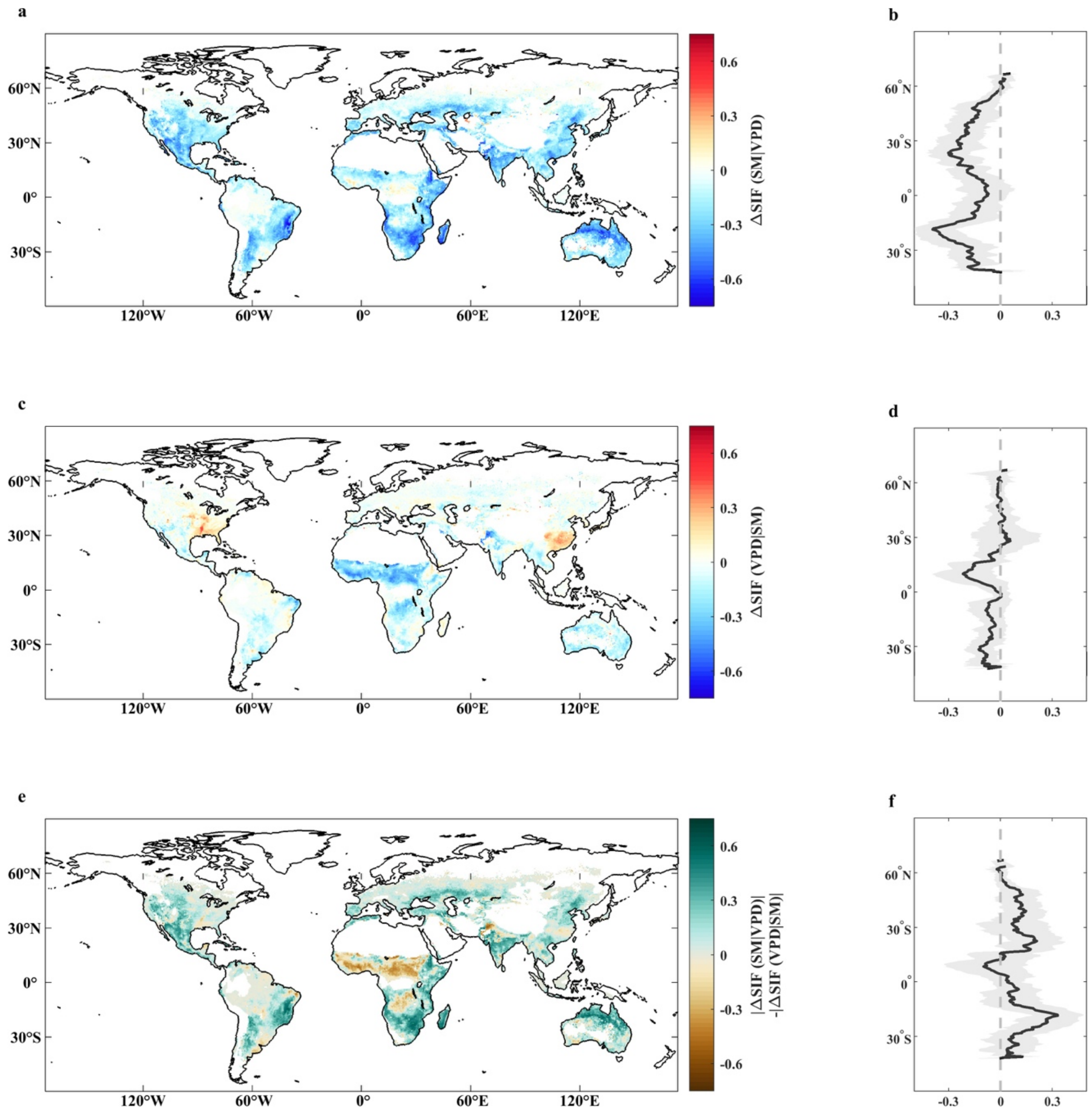

Supplementary Figure 12. Same as Fig. 4, but removing possible radiation effects by standardizing SIF by PAR. Black lines indicate the mean values, and gray shaded bands show the standard deviation.

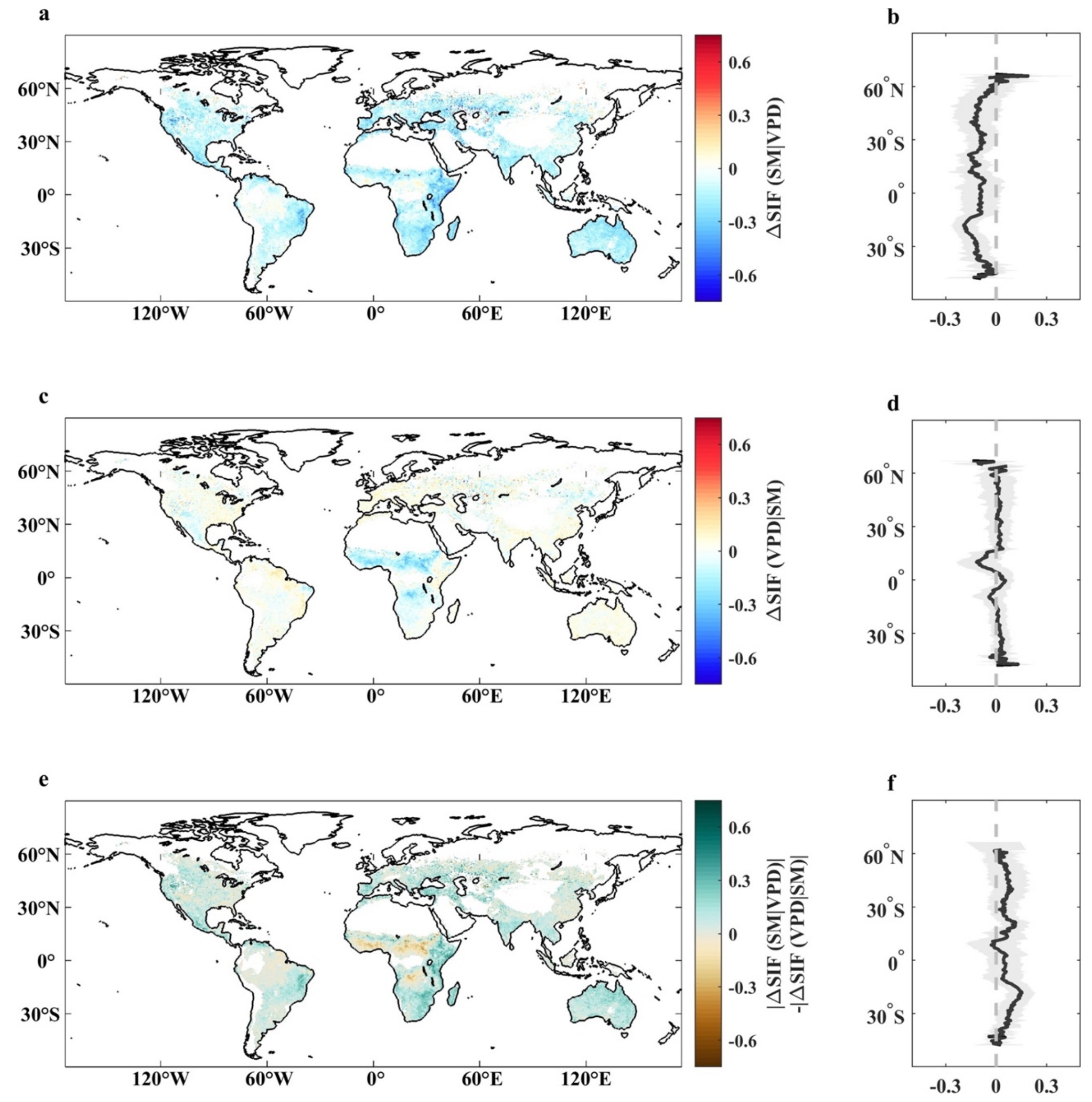

Supplementary Figure 13. Same as Fig. 4, but using the averaged estimates from narrow temperature ranges of 15°C-18°C, 18°C-21°C, 21°C-24°C, 24°C-27°C and 27°C-30°C and daily mean GOME-2 GFZ SIF. Black lines indicate the mean values, and gray shaded bands show the standard deviation.

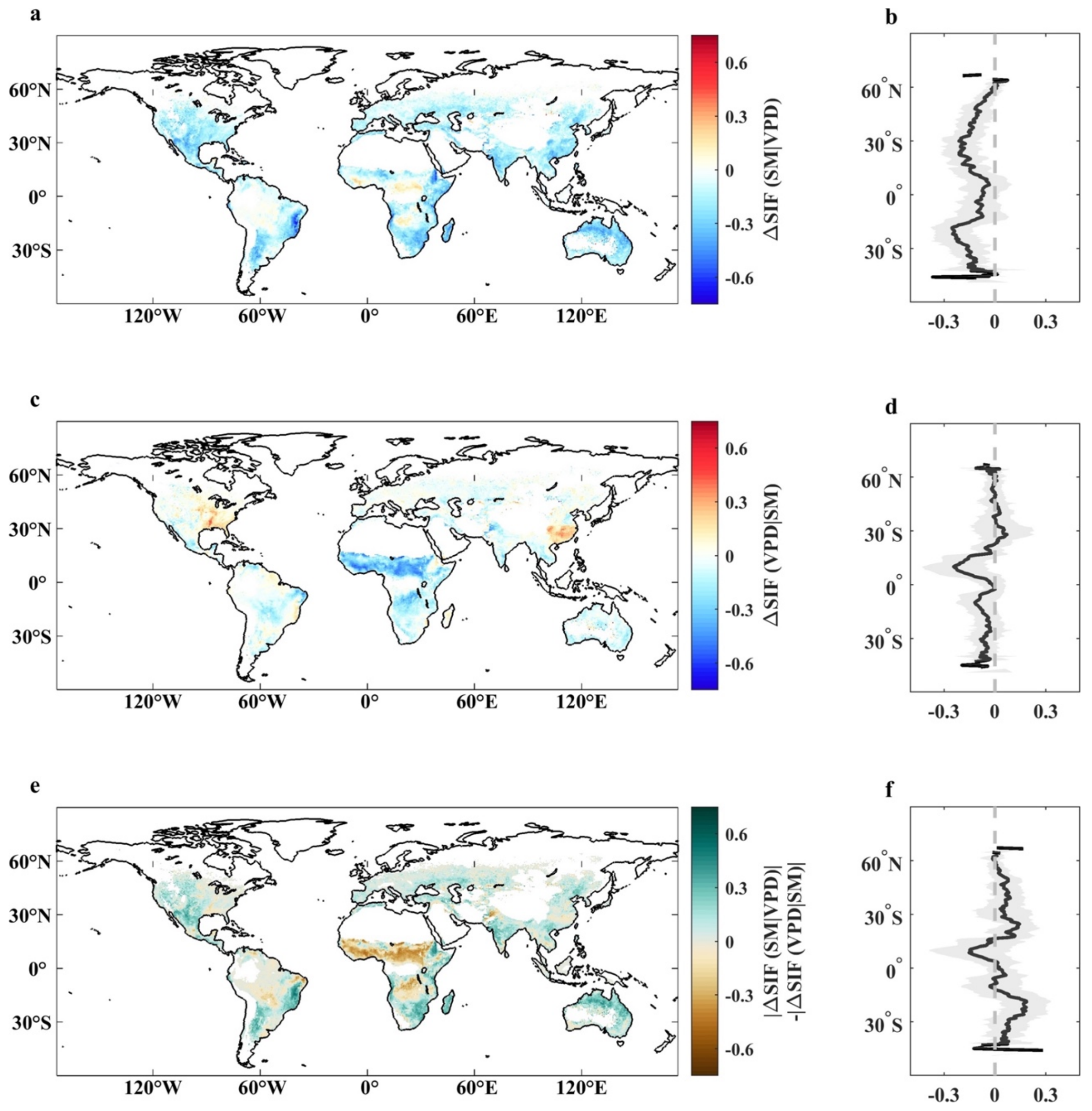

Supplementary Figure 14. Same as Fig. 4, but using the aggregated 8-days CSIF. Black lines indicate the mean values, and gray shaded bands show the standard deviation.

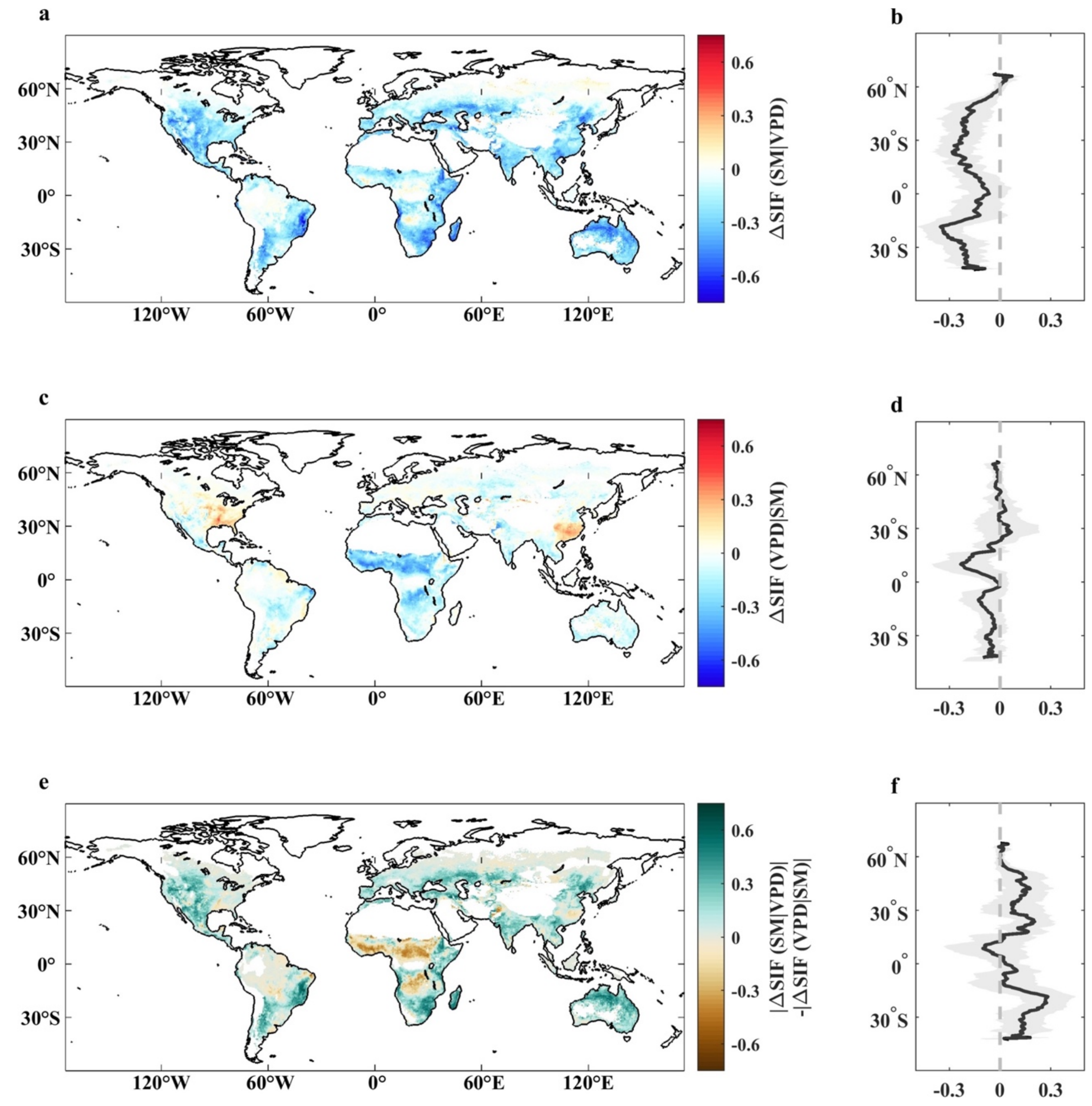

Supplementary Figure 15. Same as Fig. 4, but using 20 percentile bins. Black lines indicate the mean values, and gray shaded bands show the standard deviation.

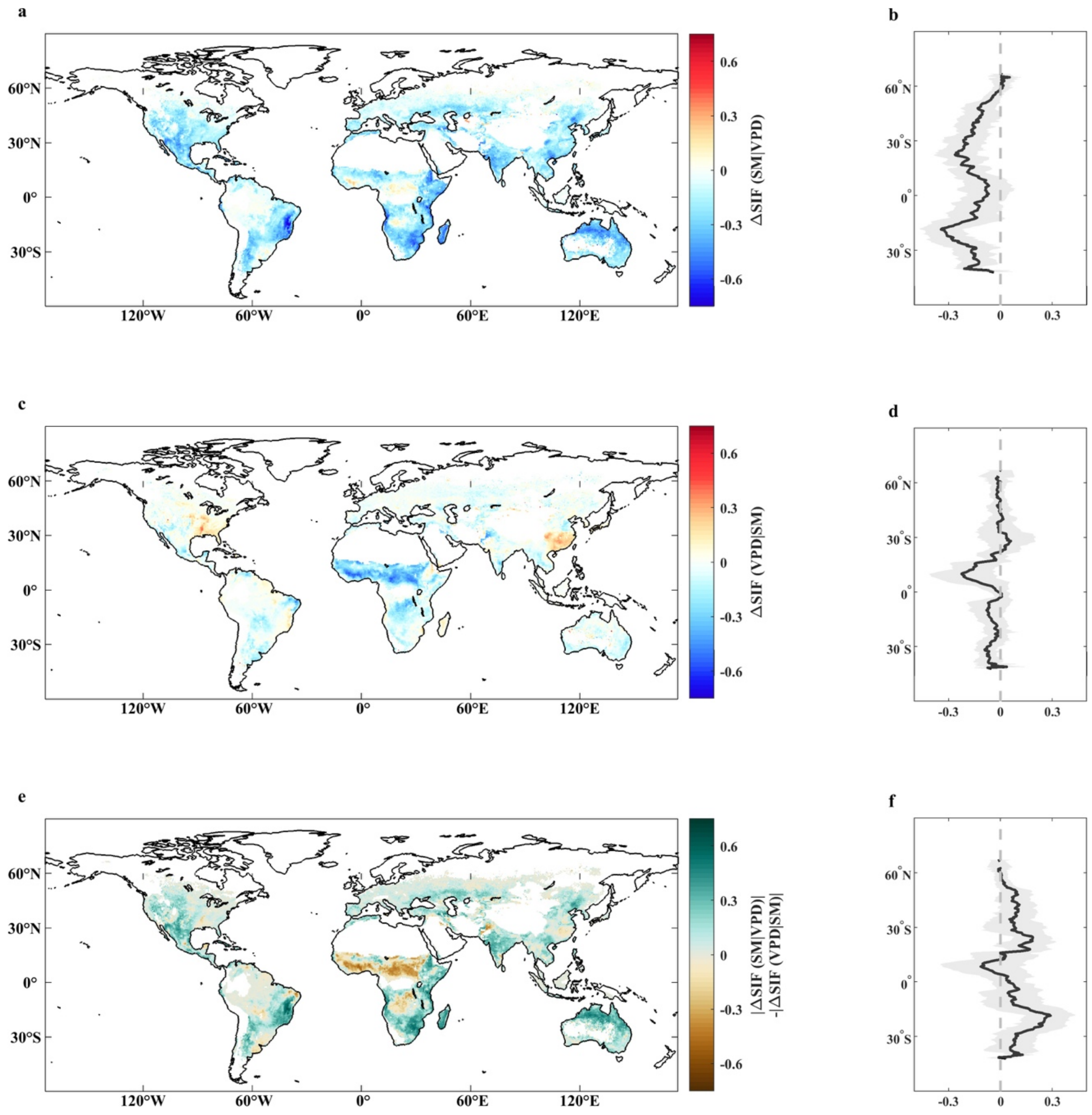

Supplementary Figure 16. Same as Fig. 4, but using the linear regression approach. Black lines indicate the mean values, and gray shaded bands show the standard deviation.

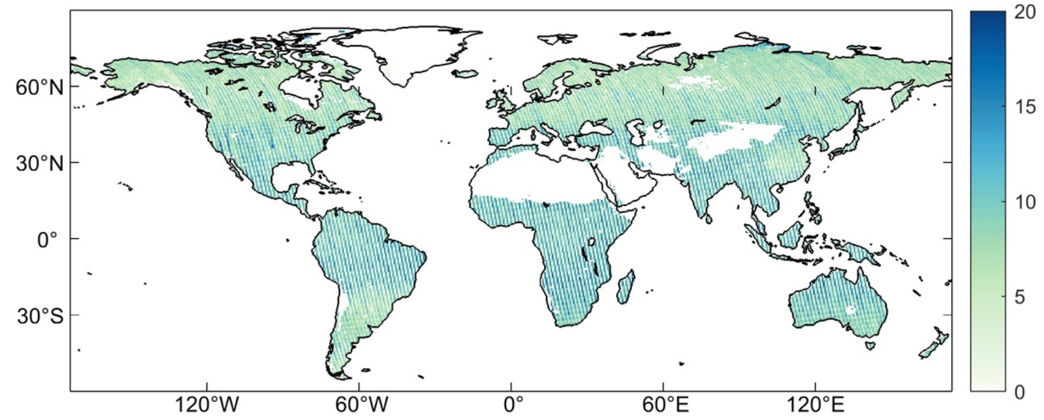

Supplementary Figure 17. Global maps of the average number of aggregated OCO-2 SIF observations after data filtering at 0.5°×0.5° per year. The aggregation only includes the OCO-2 retrievals that pass the quality criteria (documented in OCO-2 Lite files).

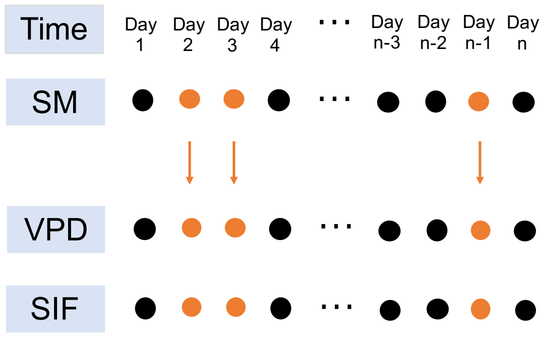

Supplementary Figure 18. Illustration of the binning procedure effect on temporal match. The orange dots indicate all data in one SM bin.

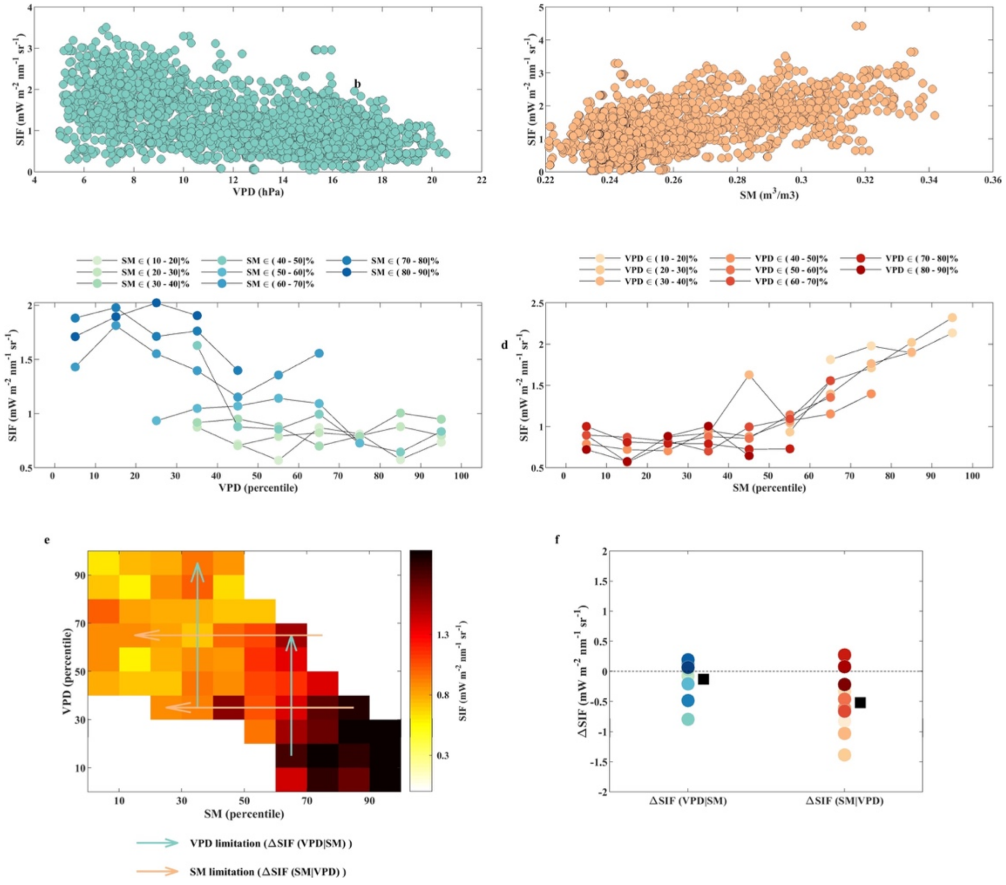

Supplementary Figure 19. Same as Figure 3, but using the pixel located at Brazil (4.25°S, 40.25°W) and GOME-2 GFZ SIF.

Supplementary Table 1. Classification of aridity regions based on aridity index.

Value	Climate Class
<0.05	Hyper Arid
0.05-0.2	Arid
0.2-0.5	Semi-arid
0.5-0.75	Sub-humid
>0.75	Humid

Supplementary Table 2. Dataset name and access links.

Dataset name	Data access
Gome-2 GFZ SIF	ftp://ftp.gfz-potsdam.de/home/mefe/GlobFluo/GOME-2/ , accessed on 12 November 2018
Gome-2 N28 SIF	https://avdc.gsfc.nasa.gov/pub/data/satellite/MetOp/GOME_F/v28/
SCIAMACHY SIF	ftp://ftp.gfzpotsdam.de/home/mefe/GlobFluo/SCIAMACHY/, accessed on 12 November 2018
OCO-2 CSIF	https://figshare.com/articles/CSIF/6387494
ERA-Interim	https://www.ecmwf.int/en/forecasts/datasets/reanalysis-datasets/era-interim
MEERA-2	https://gmao.gsfc.nasa.gov/reanalysis/MERRA-2/data_access/
ESA CCI, version 04.4	https://www.esa-soilmoisture-cci.org/index.php?q=node/145
CERES_SYN1deg_Ed4A	https://ceres-tool.larc.nasa.gov/ord-tool/jsp/SYN1degEd4Selection.jsp
Climate Research Unit, version 4.01	https://crudata.uea.ac.uk/cru/data/hrg/
Global forest change (GFC), version 1.6	https://earthenginepartners.appspot.com/science-2013-global-forest/download_v1.6.html
MODIS MCD12Q1	https://modis.gsfc.nasa.gov/data/dataprod/mod12.php
